# 造血干细胞移植后肝窦阻塞综合征研究进展

**DOI:** 10.3760/cma.j.issn.0253-2727.2021.12.020

**Published:** 2021-12

**Authors:** 璇 蔡, 晓辉 张

**Affiliations:** 北京大学人民医院血液科、北京大学血液病研究所 100044 Department of Hematology, Peking University People's Hospital, Peking University Institute of Hematology, Beijing 100044, China

肝窦阻塞综合征（sinusoidal obstruction syndrome, SOS）既往称为肝小静脉闭塞病（veno-occlusive disease, VOD）是造血干细胞移植（HSCT）后的严重并发症，其临床主要表现是肝脏肿大、右上腹痛、黄疸和腹水等[1]。轻度患者病程有自限性，重症患者多合并多脏器功能不全综合征（MODS）或多器官功能衰竭（MOF）[2]。本文对成人HSCT后SOS/VOD的发病机制、危险因素、诊断标准和治疗进展等进行回顾。

一、流行病学

由于不同的患者群体、预处理方案、移植类型、危险因素以及诊断标准的差异等因素，HSCT后SOS/VOD的发生率为9.6％～17.3％[4]–[6]。随着HSCT各项技术的改进和支持治疗的完善，SOS的发生率呈下降趋势，预后显著改善[3]–[4]。近期研究报告表明，SOS/VOD患者移植后100 d生存率超过50％[5]–[6]。

二、发病机制

目前SOS的发病机制尚未完全明确。研究表明，最早及最重要的病理改变是肝窦内皮细胞损伤、细胞间连接被破坏，红细胞等进入内皮细胞下Disse间隙，导致内皮细胞从基底膜脱落，造成血窦阻塞等[7]。凝血-纤溶系统失衡可能导致微血管内血栓形成，进一步加重肝血管阻塞[8]。

三、危险因素

SOS的危险因素主要包括移植相关因素、疾病相关因素和肝脏相关因素。移植相关危险因素包括清髓性预处理方案、二次或多次HSCT、非亲缘供者或HLA不相合供者以及应用钙调磷酸酶抑制剂等[9]。研究表明，单倍型移植并未增加发病风险[10]。高龄、合并代谢综合征、原发病复发或第二次及以上完全缓解（≥CR2）患者是SOS发病的高危群体[11]。移植前肝病史、肝毒性药物用药史、腹部或肝脏放疗史、病毒性肝炎及肝铁过载是其发病的危险因素[12]。

四、影像学及其他检查

超声、CT和磁共振（MRI）是常用的SOS相关影像学检查。超声检查可持续评估病情进展，早期超声检查可见肝脏弥漫性肿大、肝内斑片状低回声、胆囊壁增厚和腹水[13]–[14]，后期出现较高特异性的肝脏血流动力学异常（门脉高压、门静脉血流速度减慢或逆向血流）[15]–[17]。SOS患者的肝脏弹性值（LSM）显著增加，有效治疗后LSM减低，因此LSM是预测发病和监测治疗反应的指标[17]–[18]。肝脏组织病理学是诊断的金标准，但血小板减少和（或）凝血功能异常限制了肝脏活检的应用[19]。

五、诊断及分级标准

成人SOS常用的临床诊断标准包括1987年Jones等制定的巴尔的摩标准[20]、1993年MaDonald等基于西雅图标准修订的改良西雅图标准[21]以及2015年欧洲骨髓移植学会（EBMT）制定的EBMT标准[1]。

巴尔的摩标准：HSCT后21 d内出现高胆红素血症（血清胆红素≥20 mg/L），在此基础上，具备以下3条中至少2条：肝大伴疼痛、体重增加>5％、腹水。

改良西雅图标准：HSCT后20 d内出现以下3条中至少2条：血清胆红素≥20 mg/L、肝大或右上腹痛、体重增加>2％。

EBMT标准：（1）HSCT后21 d内起病的患者，保留巴尔的摩标准用于诊断；（2）HSCT后21 d后起病的患者，需满足以下任意1条：①21 d后起病且满足巴尔的摩标准的其他条件。②肝活检病理证据：早期表现为终末肝静脉内膜水肿、小叶中央淤血及肝细胞坏死，晚期可见肝小叶中心纤维化、终末肝静脉壁增厚和管腔狭窄或闭塞等[22]。③血流动力学和（或）超声证据（门脉血流速度减慢或逆向血流、肝大和腹水）；此外，患者需满足以下4条中至少2条：血清胆红素≥20 mg/L、肝大伴疼痛、体重增加>5％和腹水。

明确诊断后，需要评估患者病情严重程度。2016年，EBMT对疾病严重程度制定了新的分级标准（表1），主要指标包括：起病至确诊时间、胆红素水平及动态变化、转氨酶、血清肌酐和体重增加程度，分级应满足至少2个指标。随着危险因素增多，病情加重的风险增加，因此EBMT建议当患者存在至少2个危险因素时，轻度SOS应升级为中度，中度升级为重度。分级标准还可用于尚未确诊的患者，特别是在HSCT后3周内，重度或极重度的疑似患者也需要及时干预。既往临床分级标准中，轻度患者病情可自发缓解，中度患者经积极治疗能好转，100 d内未缓解或死亡的为重度患者。由于是回顾性临床评估，该标准对预后评估尤其是指导治疗具有局限性[2]。

**表1:** 欧洲骨髓移植学会（EBMT）2016年制订的SOS分级标准

临床指标	轻度	中度	重度	极重度
起病至确诊时间（d）	>7	5～7	≤4	−
胆红素（mg/L）	20～<30	30～<50	50～<80	≥80
胆红素水平动态变化	−	−	48 h内倍增	−
转氨酶（AST、ALT）（×ULN）	≤2	>2～5	>5～8	>8
体重增加	<5％	5％～<10％	5％～<10％	≥10％
血清肌酐（mg/L）	≤12	12～<15	15～<20	≥20或合并MOD/MOF
危险因素调整	−	轻度+ ≥2个危险因素	中度+ ≥2个危险因素	−

注：SOS：肝窦阻塞综合征；VOD：肝小静脉闭塞病；MOD：多脏器功能不全；MOF：多脏器功能衰竭；ULN：正常值上限；−：不适用

SOS病情转归的评估：CR是指无不适症状，腹胀、右上腹痛缓解，肝肿大、腹水等征象消失，体重、腹围恢复至基线水平，血清总胆红素<20 mg/L以及器官功能恢复正常（血清肌酐恢复至基线值1.5倍以下且不需透析，肾功能恢复正常，血氧饱和度>90％且无需吸氧或呼吸机辅助通气，肺功能恢复正常，脑病症状完全消失，中枢神经系统功能恢复正常）[23]–[24]。

六、预测标志物的研究进展

目前尚无公认的血清学预测标志物。近期研究表明，患者起病前数天至数周即可出现多种指标异常，动态监测相关指标有利于早期识别高危人群[25]。SOS患者处于高凝状态，多项研究证实起病前可见纤溶酶原激活物抑制因子1（PAI-1）水平增高，有效的治疗可使其水平降低[26]。蛋白C及抗凝血酶Ⅲ水平减低、组织纤溶酶原激活剂（t-PA）水平升高等其他凝血异常可作为预测病情的指标[27]–[28]。基于肝窦内皮损伤这一主要病理基础，联合检测其相关标志物如肿瘤抑制因子-2、血管生成素-2、血管性血友病因子（VWF）、血栓调节蛋白（TM）、纤维胶凝蛋白、血管细胞黏附分子1（VCAM-1）和细胞间黏附分子1（（ICAM-1）等可能有助于预测SOS的发生[27]–[28]。

七、治疗

SOS病情进展快、预后差，因此需要早期诊断、及时治疗，最大限度减轻肝损伤和继发多脏器功能障碍，主要受损器官包括肺、肾和中枢神经系统等[29]。

1. 支持治疗：支持治疗是SOS最基础和最重要的治疗措施，维持液体平衡尤其重要。①密切监测体重和出入量，严格限制水钠摄入，利尿减轻液体负荷，维持体重增幅在2％～5％以内；②输注白蛋白、血浆和红细胞等维持有效血容量，避免缺氧导致肝窦内皮损伤加剧，同时保留有效肾灌注，防止肝肾综合征的发生；③大量胸/腹水的患者应考虑行胸腔/腹腔穿刺引流，减轻患者不适并改善呼吸功能。个性化的综合治疗策略还包括避免或停用可导致血管内皮损伤和肝肾功能损害的药物（如钙调磷酸酶抑制剂）以及预防性抗感染治疗、营养支持和镇痛等[30]–[31]。

绝大多数轻度患者经过对症治疗可治愈，中度患者支持治疗基础上需密切监测病情变化，症状未见改善或者持续进展的中度患者以及重度或极重度患者需及时开始去纤苷治疗[26],[32]–[33]。

2. 去纤苷（Defibrotide）：去纤苷是一种多聚脱氧核糖核苷酸，是目前公认治疗SOS的有效药物。凝血和纤溶失衡是发病的重要因素，去纤苷可抑制PAI-1和VWF的活性，促进内皮细胞t-PA和TM的表达，因此减低内皮细胞活化和增强纤溶活性[34]。

不同剂量去纤苷治疗方案的安全性和有效性研究表明，25 mg·kg^−1^·d^−1^剂量组的有效率高于低剂量组（10 mg·kg^−1^·d^−1^），与高剂量组（40、60、80 mg·kg^−1^·d^−1^）相比无明显差异，而高剂量去纤苷的不良反应较25 mg·kg^−1^·d^−1^剂量组更多见，因此推荐25 mg·kg^−1^·d^−1^为临床治疗剂量[26]。早期开始去纤苷治疗可防止病情加重，而延迟治疗可使有效率和生存率明显降低[35]。53％～60％的患者需治疗21 d后才能获得CR，CR患者的100 d生存率为90％～92.5％，而治疗无效患者的100 d生存率仅为15％～37.3％，且疗程延长并未增加不良反应发生率，因此目前认为疗程不应少于21 d，可持续用药至获得CR[32]。

去纤苷的总体CR率为36％～76％，100 d生存率为32％～79％，其中未发生MODS/MOF患者的100 d生存率为64％～81％[36]。重度或极重度患者仅予支持治疗时，100 d死亡率高达70％～85％；去纤苷治疗合并MODS/MOF患者的CR率为25％～55％，100 d生存率为28％～54％[37]–[38]。HSCT后21 d内起病或HSCT后21 d后起病不影响去纤苷治疗SOS的疗效[39]。去纤苷具有良好的耐受性，不良反应多为轻度出血和低血压[5],[33],[40]。

3. 糖皮质激素：糖皮质激素具有强效的非特异性抗炎作用。前瞻性研究表明0.5 mg·kg^−1^·d^−1^甲泼尼龙治疗SOS的有效率为63％，100 d生存率为58％[41]。其他研究表明，甲泼尼龙（500 mg/m^2^每12 h 1次×3 d）的有效率为64.0％～66.7％，重症患者的有效率为40.0％[42]。血糖升高和高血压是糖皮质激素的常见不良反应，巨细胞病毒感染、血流感染和真菌感染的发生率未见明显增加[41]。其疗效尚需要大样本随机临床试验进一步验证。

4. 其他治疗：肝素和t-PA治疗有效率低，且增加出血风险，目前不推荐使用[43]。经颈静脉肝内门体分流术能降低门脉压力，但不能改善预后，不建议作为常规治疗。对于重症及药物治疗无效的肝衰竭患者，可考虑试用肝移植等。

八、预防

由于目前治疗措施有限，而且重症患者预后差，因此需要采用预防治疗以减少SOS的发生。预防措施主要包括纠正危险因素和药物预防。

去纤苷作为治疗手段的有效性促进了其作为预防用药的研究。一项包含1230例患者的Meta分析结果显示，去纤苷预防组的SOS发病率为4.7％，显著低于对照组的13.7％，且重度患者明显减少[44]。去纤苷预防治疗可降低高危患者的SOS发病率和SOS相关死亡率，且未见严重不良反应或重度出血[45]。尽管缺乏前瞻随机试验，目前的研究在一定程度上证实了去纤苷预防成人SOS的安全性和有效性，有高危因素的患者建议使用去纤苷预防[46]。其预防用药的剂量、疗程和时机需要进一步探索。

其他药物用于预防也有报道，临床病例报告熊去氧胆酸具有预防作用，但缺乏大规模研究数据支持[47]。肝素、前列腺素E1预防用药并未降低SOS的发病率，而且增加了出血风险及不良反应发生率[47]–[48]。

九、结论和展望

尽管SOS的诊疗取得了良好进展，其仍然是影响HSCT预后的重要合并症之一，优化诊疗措施对提高患者的生存率十分重要。随着HSCT技术不断进步，患者和疾病特征发生了很大改变，需要全面评估SOS的危险因素，制定高危患者的判定标准，针对不同危险程度患者制定相应的预防策略。部分患者起病于HSCT后3周后至100 d，且可无黄疸表现，需要合理制定院外随访策略，以便及时明确诊断。识别潜在特异的血清学标志物和影像学指标，有望提高诊断标准和分级标准的准确性。去纤苷预防用药的研究多为回顾性，证据级别有限，需要前瞻性临床试验进一步验证。
